# Effect of Weight on the Resonant Tuning of Energy Harvesting Devices Using Giant Magnetostrictive Materials

**DOI:** 10.3390/ma11040581

**Published:** 2018-04-10

**Authors:** Kotaro Mori, Tadashi Horibe, Shigekazu Ishikawa

**Affiliations:** 1 Department of Mechanical Engineering, College of Engineering, Ibaraki University, 4-12-1 Nakanarusawa-cho, Hitachi 316-8511, Japan; tadashi.horibe.mech@vc.ibaraki.ac.jp; 2 Hitachi Power Solutions Co., Ltd., 3-2-2 Saiwai-cho, Hitachi 317-0073, Japan; shigekazu.ishikawa.fs@hitachi.com

**Keywords:** cantilever, magnetostrictive material, energy harvesting, self-tuning

## Abstract

This study deals with the numerical and experimental study of the effect of weight on the resonant tuning and energy harvesting characteristics of energy harvesting devices using giant magnetostrictive materials. The energy harvesting device is made in a cantilever shape using a thin Terfenol-D layer, stainless steel (SUS) layer and a movable proof mass, among other things. In this study, two types of movable proof mass were prepared, and the device was designed to adjust its own resonant frequency automatically to match external vibration frequency in real time. Three-dimensional finite element analysis (FEA) was performed, and the resonant frequency, tip displacement, and output voltage in the devices were predicted and measured, and the simulation and experiment results were compared. The effects of the weight of the proof mass on self-tuning ability and time-varying behavior were then considered in particular.

## 1. Introduction

Magnetostrictive materials show elongation or contraction under external magnetic fields, and the magnetization changes in connection with applied forces [1,2]. Until now, magnetostrictive materials have been mainly used as sensors and actuators because of their suitable properties, such as high energy density, quick response and the possibility of remote operation. Recently, magnetostrictive Fe–Co. alloys have gained attention owing to their low cost and abundance compared to existing magnetostrictive materials, and the range of uses of magnetostrictive materials is expected to expand [3,4,5]. In particular, the use of these materials for energy harvesting has gained the interest of researchers, and some results in this regard have been reported [6,7]. 

Energy harvesting is defined as the conversion of the ambient energy present in the environment into usable electrical energy and is expected to be employed as the power source for devices, such as IoT (Internet of Things) devices and sensors [8,9]. In particular, devices for vibration energy harvesting are anticipated to be a substitute for battery-powered systems. Moreover, their lifetime is generally several times that of a battery, and they are a tempting substitute in vibration-rich environments. However, the primary issue in vibration energy harvesting is that the best performance of a generator is usually limited to excitation at its fundamental resonance frequency, and usually, ambient vibrations comprise several frequency components [10]. If the resonant frequency of devices deviates slightly from the frequency of the ambient vibration, the power output is drastically reduced. Thus, a major challenge for the accomplishment of energy harvesting using resonance is widening the range of the resonant frequency of the device. Over the past few years, due to the importance of resonant frequency matching to allow the generation of power from ambient vibrations, researchers have adopted various techniques to tackle this [11,12]. Firoozy et al. investigated the mechanical behavior of a broadband energy harvesting system comprising a unimorph piezoelectric cantilever beam with a tip magnet and two external permanent magnets [13]. Miller et al. considered the beam-mass resonator system, which consists of a fixed-fixed beam and a mass that is able to slide along the beam and showed that these resonators enable adjustment to varying input frequencies and thereby, increase the energy harvested over time [14]. A previous study demonstrated that the output voltage increases with the sliding of the movable proof mass, and the tendency of this movement depends on the driving frequency [15]. 

In this paper, we discuss the influence of weight on the self-tuning and power output tendencies of a magnetostrictive energy harvesting device. We adopted a combined numerical and experimental approach in this study. The energy harvesting device constituted a perforated cantilever beam and a movable proof mass installed in the slit hole of the device. Finite element analysis (FEA) was executed to forecast the resonant frequency, tip displacement, and output voltage in the device. The resonant frequency, tip displacement, output voltage, and self-tuning behavior were also estimated, and the obtained results were then compared with numerical simulation results. Then, we determined the influence of the weight of the movable proof mass based on the inclination of the proof mass to move and the principle that the direction of sliding of the proof mass changes with the input frequency.

## 2. Analysis 

### 2.1. Basic Equation

Consider the orthogonal coordinate system with axes *x*_1_, *x*_2_ and *x*_3_. The basic equations for magnetostrictive materials are as follows [16]:

(1) $$\sigma_{ji,j}=\rho u_{i,tt}+cu_{i,t}$$

(2) $$B_{i,i}=0$$

where *σ_ij_* and *B_i_* are the stress and magnetic flux density, respectively; *u_i_* is the displacement; and *ρ* and *c* are the mass density and the damping coefficient, respectively. A comma followed by an index denotes partial differentiation of the space coordinate, *x_i_*, or the time, *t*. We introduced the summation convention for repeated tensor indices. The constitutive equations can be noted by the following equations [2,17]:

(3) $$\varepsilon_{ij}=s_{ijkl}^{H}\sigma_{kl}+d_{kij}^{m}H_{k}$$

(4) $$B_{i}=d_{ikl}^{m}\sigma_{kl}+\mu_{ik}H_{k}$$

where *ε_ij_* and *H_i_* are the strain and the magnetic field intensity; *s^H^_ijkl_*, *d^m^_kij_*, *μ_ij_* are the elastic compliance for a constant magnetic field, the piezo-magnetic constant and the magnetic permittivity. The valid symmetry conditions for the material constants are as follows:

(5) $$s_{ijkl}^{H}=s_{jikl}^{H}=s_{ijlk}^{H}=s_{klij}^{H},d_{kij}^{m}=d_{kji}^{m},\mu_{ij}=\mu_{ji}$$

The strain and magnetic field intensity are related to the displacement and magnetic potential (*ϕ*) by the following relationship:

(6) $$\varepsilon_{ij}=\frac{1}{2}(u_{j,i}+u_{i,j})$$

(7) $$H_{i}=\phi_{i}$$

### 2.2. Model

**Figure 1:**
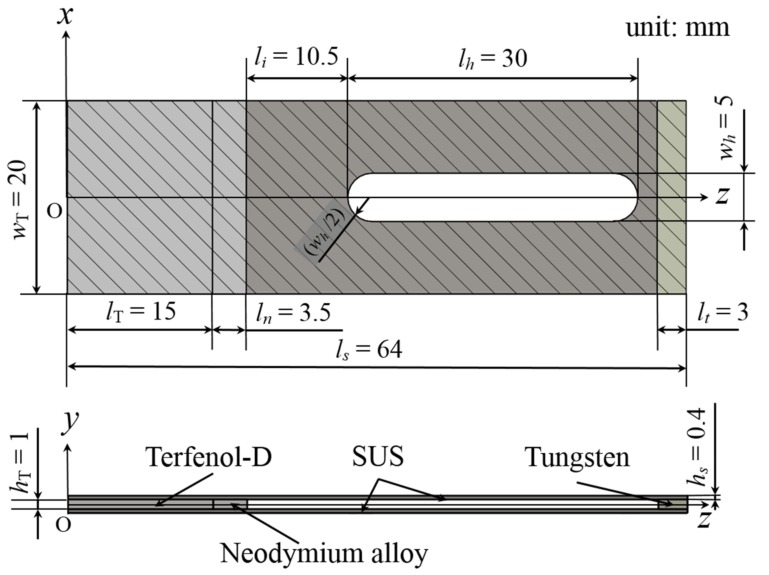
Illustration of the used energy harvesting device.

**Figure 2:**
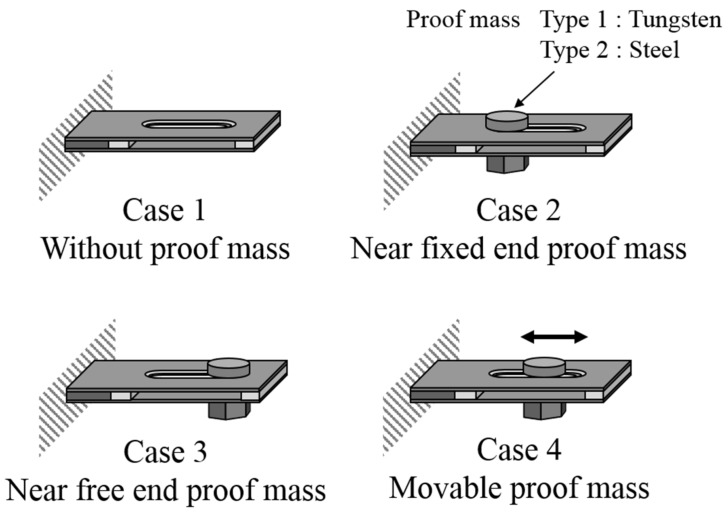
Schematic of a device with a proof mass.

The energy harvesting device used for this study comprised a cantilever with a slit hole (Figure 1). Let the coordinate axes, *x* = *x*_1_ and *z* = *x*_3_, be chosen such that they coincide with the center of the thickness direction of the device; the *y* = *x*_2_-axis is perpendicular to this plane. The origin of the coordinate system is located at the center of the fixed end of the device. The Terfenol-D layer is magnetized in the *z*-direction. The Terfenol-D, neodymium, and tungsten layers are sandwiched between two perforated stainless steel (SUS) layers. Here, *l*, *w* and *h* are the length, width and thickness, respectively, and the subscripts, *T*, *n*, *t* and *s* indicate the Terfenol-D, neodymium, tungsten and SUS layers, respectively. A slit hole (length *l_h_* and width *w_h_*) was created on the SUS layer at a distance, *l_i_*, from the neodymium layer. Let us now consider three cases of mass position and two mass types, as shown in Figure 2 (Cases 1–3 and Types 1 and 2). The imposed base excitation is applied by the displacement, *u_y_*_0_ exp(*iϖt*), of the fixed end (*z* = 0 plane), where *u_y_*_0_ is the amplitude of the applied displacement and *ϖ* is the angular frequency. The damping ratio determined from the experiments was adopted in the model. First, we calculated the tip displacement using FEA to gain the resonant frequency of each case and type. Then, we performed FEA for the induced magnetic field to obtain the output voltage. The output voltage was calculated using the induced magnetic field by following Faraday’s law:

(8) $$V_{out}=-Nw_{T}h_{T}\frac{dB_{z}}{dt}$$

where *N* is the turn number of a search coil; *B_z_* is the *z*-component of magnetic induction; and *t* is the time. The constitutive equations for magnetostrictive materials are mathematically equivalent to those for piezoelectric materials. Therefore, coupled-field solid elements in ANSYS were used in the analysis. Only half of the device model was fabricated. In total, 7080 nodes and 15,161 elements and 12,285 nodes and 18,234 elements were adopted for Case 1 and Cases 2 and 3, respectively (there was no difference in the number of elements by type). The finite element calculations were performed by correcting the program with routines developed in our past work [15], and mesh sensitivity was established—the mesh was fine enough. 

## 3. Experiments 

The energy harvesting device was constructed by sliding the Terfenol-D, neodymium, and tungsten layers between two perforated SUS layers, as shown in Figure 1. The dimensions of the Terfenol-D layer were as follows: length *l_T_* = 15 mm, width *w_T_* = 20 mm, and thickness *h_T_* = 1 mm. The dimensions of the SUS layer were as follows: length *l_s_* = 64 mm, width *w_s_* = 20 mm, and thickness *h_s_* = 0.4 mm. The lengths of the neodymium and tungsten layers were *l_n_* = 3.5 mm and *l_t_* = 3 mm, respectively. The Terfenol-D, neodymium, and tungsten layers were perfectly glued between two SUS layers using epoxy. The gap between the neodymium and the slit hole was *l_i_* = 10.5 mm, and the slit hole (*l_h_* = 30 mm, *w_h_* = 5 mm) existed at the center of the SUS layer (at (*l_T_* + *l_n_* + *l_i_* + *l_h_*/2) = 44 mm from the fixed end). We considered four cases of mass position (Cases 1–4) and two mass types (Types 1 and 2), as shown in Figure 2. The masses of Types 1 and 2 were made of tungsten and steel. Table 1 and Table 2 specify the materials’ properties. The movable proof mass was deployed in the slit hole, and it was tightened at the corner on the free-end side or the fixed-end side of the hole of the device (Cases 2 and 3, respectively). 

First, we measured the tip displacement (*u_y_*) for the device using a laser displacement meter (LK-G30; Keyence Corporation, Osaka, Japan). Sinusoidal vibration was generated by the following equipment: a function generator (DF1905; NF Corporation, Yokohama, Japan), a vibration shaker (ET-132; Labworks Inc., Portland, OR, USA), and an amplifier (PA-151; Labworks Inc.). The specimen was mounted on the shaker and vibrations were applied. The input frequency, *f* = *ϖ*/2*π*, was altered between 10 and 200 Hz at intervals of 10 Hz (except near the resonant frequency, around which 1 Hz intervals were employed). At each frequency, the generator amplitude was adjusted to an acceleration of *a* = *u_y_*_0_*ϖ*^2^ = 1g. 

**Figure 3:**
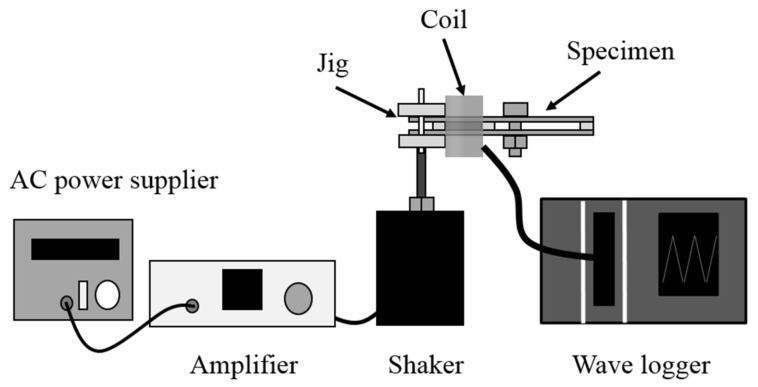
Experimental setup for measuring the output voltage.

Next, the output voltage (*V_out_*) of the device was measured using a search coil and a wave logger (NR-600; Keyence Corporation, Osaka, Japan) (see Figure 3). The coil had a rectangular cross section with dimensions 22 × 2.5 mm^2^ and a length of 20 mm. It was wound using an 80 μm diameter-enameled copper wire and had 2300 turns. We removed the constraint of the proof mass, enabling mobility of the mass (Case 4), and then measured the time-varying output voltage, *V_out_*. The movable proof mass could freely slide within the slit hole, and the self-tuning behavior was examined under a high-speed microscope (VW-9000; Keyence Corporation, Osaka, Japan). 

## 4. Results and Discussion

**Figure 4:**
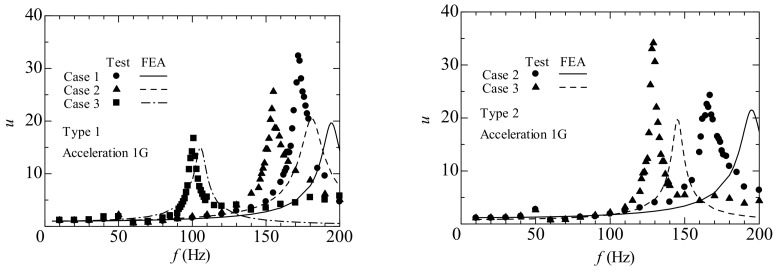
Normalized displacement versus frequency for the energy harvesting devices (Types 1 and 2).

**Figure 5:**
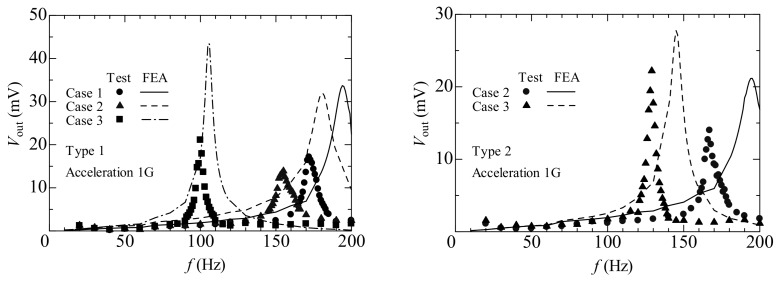
Output voltage versus frequency for the energy harvesting devices (Types 1 and 2).

We first discuss the results of Cases 1, 2, and 3. Figure 4 shows the normalized displacement, *u_y_*/*u_y_*_0_, as a function of frequency (*f*) for energy harvesting device Types 1 and 2. Here, *u_y_*_0_ is the displacement of the shaker. The lines and plots stand for the results of the FEA and experiment, respectively. Here, the lines are the results pertaining to the damping ratio obtained using the fast Fourier transform (FFT) analyzer (CF-7200; Ono Sokki Corporation, Yokohama, Japan) [15]. The damping ratios (*ζ*) of Cases 1, 2, and 3 of Type 1 (Type 2) were 0.0311, 0.0374 (0.0364), and 0.0225 (0.0252), respectively. The measured resonant frequencies of Cases 1, 2, and 3 of Type 1 were approximately 172, 155, and 101 Hz, respectively, and the calculated resonant frequencies were approximately 194, 181 and 105 Hz, respectively. The measured resonant frequencies for Cases 2 and 3 of Type 2 were approximately 167 and 129 Hz, and the calculated resonant frequencies were approximately 195 and 145 Hz, respectively. In Types 1 and 2, a reduction in resonant frequency was observed because of the existence of the proof mass, and the resonant frequency shift with change in the location of the proof mass. The results for Type 1 indicate an agreement between the experiment and analysis compared to the results of Type 2. Figure 5 shows the output voltage (*V_out_*) as a function of frequency (*f*) for energy harvesting devices Types 1 and 2 under the open-circuit condition, as obtained from the FEA and experiment. The maximum output voltages for Cases 1, 2, and 3 of Type 1 (Type 2) were approximately 17.2, 13.9 (14.0), and 21.1 (22.2) mV, respectively. The output voltage and displacement noted analogous tendencies with regard to the resonant frequency. 

**Figure 6:**
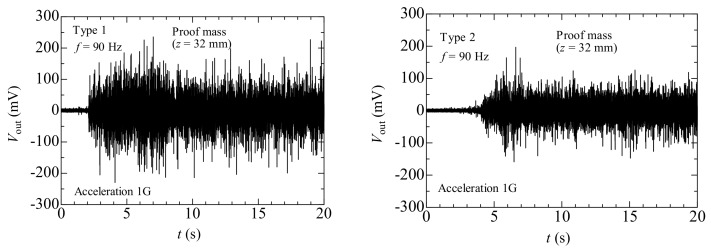
Output voltage versus time for the energy harvesting device with the proof mass (*f* = 90 Hz, Types 1 and 2).

Next, the results of Case 4 are presented. Table 3 classifies the tendency for proof mass movement corresponding to each frequency and type of weight. We estimated three starting positions: the fixed-end side (*z* = 32 mm), the free-end side (*z* = 56 mm), and the center of the hole (*z* = 45 mm). Types 1 and 2 both demonstrated that the tendency for proof mass movement reverses with 50 Hz as the border. At frequencies lower than 50 Hz, the proof mass slid from the free-end towards the fixed-end. However, at frequencies exceeding 50 Hz, the proof mass types demonstrated different movement tendencies. In Type 1, the proof mass moved from the fixed-end towards the free-end at frequencies exceeding 60 Hz. Further, at frequencies above 140 Hz, the proof mass stopped on the way. Figure 6 shows the output voltages (*V_out_*) of Type 1 and Type 2 versus time (*t*) for the energy harvesting device with the movable proof mass at *f* = 90 Hz (the starting position is fixed-end side). The output voltage increased with proof mass movement regardless of proof mass type, staying position and input frequency, and showed a similar waveform to Figure 6 (not shown here). In addition, the output voltage amplitude in Case 4 was more than 10 times that in Cases 1, 2, and 3. Nevertheless, the increase in voltage associated with the movement of the proof mass of Type 1 was larger than that observed for Type 2. This difference is attributed to the difference in weight; in other words, the vibration of the heavier proof mass resulted in a larger force on the magnetostrictive layer. In addition, it is interesting to note that the output voltage showed a tendency similar to that seen in Figure 6 when the proof mass moved in the opposite direction. In order to clarify the capability of increasing the output voltage through self-tuning, we investigated the physical phenomenon of proof mass movement with different external frequencies. 

Here, in order to clarify the phenomenon of change in the movement of the proof mass with the input frequency, we considered the relationship between the tendency for proof mass movement and tip displacement. Table 4 and Table 5 list the relationship between the tendency for proof mass movement and tip displacement of Types 1 and 2. From Table 4, we can observe that when the tip displacement exceeds 0.17 mm, the proof mass moves to the fixed-end side. Conversely, when the tip displacement is between 0.045 mm and 0.17 mm, the proof mass moves to the free-end side, and when it is less than 0.045 mm, the proof mass does not move. From Table 5, we can observe that when the tip displacement exceeds 0.06 mm, the proof mass moves to the fixed-end side. Conversely, when the tip displacement is between 0.045 mm and 0.06 mm, the proof mass moves to the free-end side, and when it is less than 0.045 mm, the proof mass does not move. It is interesting to note that the border of tip displacement that determines the direction of proof mass movement differs depending on the proof mass type. In addition, there a threshold tip displacement exists for start the process, regardless of the weight of the proof mass. Therefore, we considered the physical phenomenon of the movement by observing the proof mass under a high-speed microscope. In either movement, the proof mass moved like an inchworm, or a looper—a type of caterpillar. The proof mass was driven by two mechanisms, namely, a nut swung like a pendulum, which hauled itself by the friction between the upper surface of the cantilever and screw head. In addition, the imperceptible inclination of the screw head to the direction of movement was recognized. We also considered the cases where the proof mass stopped on the way and where the proof mass hopped on the site. The impact of a cantilever is generated by swinging the nut, and it causes an increase in the output voltage. 

## 5. Conclusions

A numerical and experimental evaluation of an energy harvesting device with resonant tuning was conducted, and the influence of weight on the resonant-tuning function was considered. The resonant frequency was found to depend on the weight and location of the movable proof mass. The trend of proof mass movement depended on the input frequency and correlated with tip displacement. In addition, the movement range and difference in tendency depended on the weight of the proof mass. For both the used weight types, at near resonant frequency, the movable proof mass stayed in a steady position or moved to an end side. However, regardless of the weight type, the output voltage increased with proof mass movement. This study may be helpful in designing progressive vibration energy harvesting devices that do not require external power to adjust their resonant frequency to the input vibration. In addition, self-tuning mechanisms using a moving proof mass are easy to combine with other wideband mechanisms (such as, using a magnet or stopper) also. Future work on self-tuning mechanisms will involve extensive inspections of the factors that initiate the movement of the proof mass and its effect on output power. 

## Figures and Tables

**Table 1:** Material properties of Terfenol-D [2,18].

	Elastic Compliance (×10^−12^ m^2^/N)					Piezo-Magnetic Constant (×10^−9^ m/A)			Magnetic Permittivity (×10^−6^ H/m)		Density (kg/m^3^)
	*s^H^*_11_	*s^H^*_33_	*s^H^*_44_	*s^H^*_12_	*s^H^*_13_	*d^m^*_31_	*d^m^*_33_	*d^m^*_15_	*^T^*_11_	*^T^*_33_	
Terfenol-D	17.9	17.9	26.3	−5.88	−5.88	−5.3	11	28	36.1	13.7	9250

**Table 2:** Material properties.

	Density (kg/m^3^)	Poisson’s Ratio (-)	Young’s Modulus (GPa)
Stainless Steel	7930	0.3	193
Neodymium magnet	7500	0.3	170
Tungsten	19,000	0.28	190

**Table 3:** Movement of the proof mass corresponding to input frequency.

	Starting Position					
	Fixed-End Side (*z* = 32 mm)		Free-End Side (*z* = 32 mm)		Middle (*z* = 44 mm)	
*f* (Hz)	Type 1	Type 2	Type 1	Type 2	Type 1	Type 2
10	×	×	○	×	○	△
20	×	×	○	○	○	○
30	×	×	○	○	○	○
40	×	×	○	○	○	○
50	×	×	○	×	○	×
60	○	○	×	×	○	○
70	○	○	×	×	○	○
80	○	○	×	×	○	○
90	○	○	×	×	○	○
100	○	×	×	×	○	△
110	○	×	×	×	○	△
120	○	×	×	△	○	×
130	○	×	×	△	△	○
140	△	×	×	○	△	○
150	△	×	×	○	△	○
160	△	×	×	○	△	○
170	△	×	×	×	△	×
180	△	×	×	×	×	×
190	×	×	×	×	△	×
200	×	×	×	×	△	×
	Toward free-end side			○: Move to the other side		
	Toward fixed-end side			△: Stop before end side		
				×: No movement		

**Table 4:** Relationship between (**a**) Tip displacement and (**b**) Movement of the proof mass corresponding to input frequency (Type 1).

(a)			(b)		
	Case 2	Case 3		Starting Position	
*f* (Hz)	*u*_tip_ (mm)		*f* (Hz)	Fixed-End Side (*z* = 32 mm)	Free-End Side (*z* = 56 mm)
10	2.745	2.782	10	×	○
20	0.645	0.684	20	×	○
30	0.330	0.368	30	×	○
40	0.233	0.301	40	×	○
50	0.222	0.188	50	×	○
60	0.039	0.041	60	○	×
70	0.047	0.059	70	○	×
80	0.048	0.072	80	○	×
90	0.048	0.086	90	○	×
100	0.050	0.378	100	○	×
110	0.049	0.124	110	○	×
120	0.051	0.074	120	○	×
130	0.061	0.061	130	○	×
140	0.085	0.043	140	△	×
150	0.164	0.039	150	△	×
160	0.156	0.042	160	△	×
170	0.080	0.034	170	△	×
180	0.058	0.037	180	△	×
190	0.041	0.034	190	×	×
200	0.030	0.034	200	×	×
	0.17 mm~		○: Move to the other side		
	0.045~0.17 mm		△: Stop before end side		
	~0.045 mm		×: No movement		
				Toward free-end side	
				Toward fixed-end side	

**Table 5:** Relationship between (**a**) Tip displacement and (**b**) Movement of the proof mass corresponding to input frequency (Type 2).

(a)			(b)		
	Case 2	Case 3		Starting Position	
*f* (Hz)	*u*_tip_ (mm)		*f* (Hz)	Fixed-End Side (*z* = 32 mm)	Free-End Side (*z* = 56 mm)
10	2.697	2.690	10	×	×
20	0.658	0.678	20	×	○
30	0.349	0.363	30	×	○
40	0.240	0.250	40	×	○
50	0.277	0.266	50	×	×
60	0.047	0.045	60	○	×
70	0.054	0.051	70	○	×
80	0.059	0.056	80	○	×
90	0.048	0.056	90	○	×
100	0.051	0.058	100	×	×
110	0.055	0.070	110	×	×
120	0.059	0.118	120	×	△
130	0.060	0.456	130	×	△
140	0.057	0.099	140	×	○
150	0.078	0.061	150	×	○
160	0.137	0.044	160	×	○
170	0.166	0.040	170	×	×
180	0.058	0.032	180	×	×
190	0.047	0.026	190	×	×
200	0.038	0.026	200	×	×
	0.06 mm~		○: Move to the other side		
	0.045~0.06 mm		△: Stop before end side		
	~0.045 mm		×: No movement		
				Toward free-end side	
				Toward fixed-end side	
